# Comparison of Signaling Pathways Gene Expression in CD34^−^ Umbilical Cord Blood and Bone Marrow Stem Cells

**DOI:** 10.1155/2016/5395261

**Published:** 2015-12-29

**Authors:** Rafał Stojko, Monika Bojdys-Szyndlar, Agnieszka Drosdzol-Cop, Andrzej Madej, Krzysztof Wilk

**Affiliations:** ^1^School of Health Sciences in Katowice, Chair of Woman's Health, Medical University of Silesia, Ulicy Medyków 12, 40-752 Katowice, Poland; ^2^Department of Obstetrics and Gynecology, The Boni Fratres Catoviensis Hospital, Ulica Księdza Leopolda Markiefki 87, 40-211 Katowice, Poland

## Abstract

The aim of the study was to compare the biological activity of the total pool of genes in CD34^−^ umbilical cord blood and bone marrow stem cells and to search for the differences in signaling pathway gene expression responsible for the biological processes. The introductory analysis revealed a big similarity of gene expression among stem cells. When analyzing GO terms for biological processes, we observed an increased activity of JAK-STAT signaling pathway, calcium-mediated, cytokine-mediated, integrin-mediated signaling pathway, and MAPK in a cluster of upregulating genes in CD34^−^ umbilical cord blood stem cells. At the same time, we observed a decreased activity of BMP signaling pathways, TGF-beta pathway, and VEGF receptor signaling pathway in a cluster of downregulating genes in CD34^−^ umbilical cord blood stem cells. In accordance with KEGG classification, the cytokine-cytokine receptor interaction, toll-like receptor signaling pathway, and JAK-STAT signaling pathway are overrepresented in CD34^−^ umbilical cord blood stem cells. A similar gene expression in both CD34^−^ UCB and BM stem cells was characteristic for such biological processes as cell division, cell cycle gene expression, mitosis, telomere maintenance with telomerase, RNA and DNA treatment processes during cell division, and similar genes activity of Notch and Wnt signaling pathways.

## 1. Introduction

In recent years the scientific environment has expressed a great interest in the nonhematopoietic stem cells (CD34^−^ and CD45^−^). These stem cells are capable of replicating* in vitro* without adding any growth factors in the period of more than 10 passages, and, when induced properly, differentiate into at least three types of mesoderm layer cells: osteoblasts, adipocytes, and chondrocytes [1, 2]. They are frequently referred to as the mesenchymal stem cells (MSCs). Due to their role in tissue repair processes their clinical potential for systemic and local transplantation procedures is significant, both as a carrier in gene therapy and for generating tissues and organs in tissue engineering procedures.

Studies published to date have stressed that the MSCs of bone marrow and of fetal origin are very similar in immunophenotypical and immunohistochemical function. The analysis of surface antigen markers by flow cytometry did not reveal any significant differences [3–5] among bone marrow and fetal MSCs. Panepucci et al. [5] showed that the MSCs of bone marrow and umbilical cord blood reveal similarities among a thousand of most expressed transcripts assayed. However, differences are seen at the molecular level in gene expression profiles of MSCs coming from different sources. For example, a distinct expression profile was characteristic for genes related to antimicrobial activity and to osteogenesis, and this distinct expression profile was more common in the MSC population from bone marrow. In the umbilical cord blood MSCs, higher expression was observed for signaling pathway genes that participate in matrix remodeling through metalloproteinases and genes related to angiogenesis. Similar results were demonstrated in studies assessing the differentiation ability in comparable* in vitro* conditions. The umbilical cord blood MSCs showed higher possibility of differentiation into osteogenic lineage and had little or no differentiation into adipocytes. This contrasted with bone marrow MSCs, where expression of markers characteristic for adipocytes was more frequently demonstrated [3, 6]. In the critical processes of regulating self-renewal and the cellular purpose, stem cells use the signaling pathways which appear to be quite conservative from the evolutionary perspective, such as Notch, Wnt, and JAK-STAT. Although the signaling proteins expression is believed to be a highly restrictive process, it appears that different stem cell types demonstrate varied rates of expression of these three families of signaling molecules. The global gene expression profile is commonly used to identify the transcription signature of specific stem cells. This signature gives insight into the signaling mechanisms regulating the self-renewal and cellular purpose program, especially in embryonic and hematopoietic stem cells. Moreover, by comparing the gene expression profiles in different stem cell groups, a common pool of genes were identified that serve either as stem cells markers for self-renewal or direct the cells through differentiation [7–11]. In comparison with a great number of studies carried out on the embryonic, hematopoietic, or neural stem cells, there are much fewer studies of molecular mechanisms of MSC self-renewal and differentiation control, mainly due to their diversified gene signature and the lack of agreement on typical markers antigens as far as some MSC markers are concerned [12–15].

This paper provides a comparison of the expression of the entire gene pool of MSC markers, with a special consideration to the signaling pathway genes in CD34^−^ stem cells which phenotypically correspond with MSCs, from the umbilical cord blood and bone marrow. The cells were extracted by means of the same single-bed room method, on the basis of the same antigen phenotype. Each cell population was multiplied three times in the same culture conditions. Gene activity was defined through the oligonucleotide microarrays with the use of GO and KEGG databases. We analyzed the nonhematopoietic stem cell signature based on the gene activity of the conservative signaling pathways, including Wnt and Notch. We then asked the question whether differences in the signaling pathways for gene activity may be evidence of different populations of origin for the MSCs (e.g., fetal verses adult origin) and consequently the predominance of one population over the other. Does a source of population, which undoubtedly shapes the cell epigenetic conformation, have a significant impact on its subsequent biological activity?

## 2. Materials and Methods

Material for the study of gene expression by means of oligonucleotide microarrays was sampled from CD34^−^ stem cell cultures from the bone marrow and umbilical cord blood. Complete RNA was extracted from the abovementioned cultures.

### 2.1. Obtaining Umbilical Blood Samples

Samples of umbilical blood were obtained after the delivery and cord clamping in the cases of normal course of pregnancy and delivery after the 37th week. The blood was obtained intranatally, that is, after omphalotomy and before placenta expulsion.

### 2.2. Obtaining Bone Marrow Samples

For the purpose of the study, approximately 6 mL of bone marrow was obtained from healthy donors during the procedure of collecting material for hematological purposes.

The research program was approved by the Bioethical Committee of the Medical University of Silesia in Katowice, Poland. All participants provided their written informed consent to participate in this study.

### 2.3. Isolation of CD34^−^ Cells from Umbilical Cord Blood and Bone Marrow

Freshly collected umbilical cord blood and bone marrow were diluted with phosphate-buffered saline (PBS Gibco), layered on Ficoll (Sigma) at 2 : 1, and centrifuged for 40 min at 400 ×g at 20°C. The lymphocytic skin obtained after centrifugation was suspended in the base with an addition of serum and centrifuged again for 10 min at 200 ×g, at 20°C. All the cell sample preparation activities were repeated twice. The prepared cells were divided into CD34^+^ and CD34^−^ fractions by means of the CD34 Progenitor Cell Isolation Kit and the Mini&MidiMACS Starting Kit. Cells viability was determined in the fluorescent microscope study after staining the cells with ethidium bromide and acridine orange.

### 2.4. CD34^−^ Cell Culture

#### 2.4.1. Cell Labeling

The cells were suspended in the appropriate volume of buffer, −0.3 mL per each 10^8^ of cells. 0.1 mL of FcR Blocking Reagent was added to the cell suspension of 3 × 10^7^/mL density, with the ratio of 0.1 mL FcR BR/0.3 mL cell suspension. For each 0.3 mL of cell suspension 0.1 mL of CD34 Microbeads was then added. The material was mixed thoroughly and incubated for 30 min at 6–12°C. 20 mL of buffer was added and mixed thoroughly again. The mixture was centrifuged for 10 min at 400 g. The supernatant was discarded and the sediment was suspended in 1 mL of buffer.

#### 2.4.2. Cell Separation in Magnetic Column

LS column was placed in the magnetic field. The column was rinsed with 3 mL of buffer, and the cell mixture as prepared above was added to the column. After the suspension had passed through the column, the CD34^−^ fraction was obtained. The column was washed with buffer (2x 0.5 mL), removed from the magnetic field, and then placed in a 5 mL tube. Next, 1 mL of buffer was applied to the column. The cells were eluted by means of a piston. In this way the CD34^+^ fraction was obtained.

#### 2.4.3. Starting the Culture

The separated CD34^−^ cell fractions were used for cell cultures. The IMDM-ISCOVE'S modified Dulbecco's medium with penicillin-streptomycin and 5% human albumin were used for culturing.

#### 2.4.4. Total RNA Extraction from Cell Culture

RNA was extracted from the CD34^−^ cell cultures with the Total RNA Prep Plus (*A&A Biotechnology*) based on the modified Chomczyński and Sacchi's method. At the initial stage, the cells were lysed, while the endogenous RNases were inactivated with fenozol. After adding chloroform, the mixture was centrifuged, and the collected RNA was precipitated with isopropanol. Next, the mixture was centrifuged for 20 min at 13 000 r/min, the supernatant was decanted, and the RNA sediment was washed twice with 75% ethanol. After drying, the sediment was stored at −70°C for the analysis.

### 2.5. Qualitative Assessment of RNA Extracts

RNA extracts underwent qualitative assessment via the agarose gel electrophoresis in 0.8% agarose gel with ethidium bromide (0.5 mg/mL) in the SUBMINI apparatus. 5 *μ*L of extract was mixed with 3 *μ*L of gel-loading dye (0.05% w/v bromophenol blue, 60% glycerol) and heated at 65°C for 4 min. After the separation, the electropherograms were assessed with the UV transilluminator (*λ* = 260 nm).

### 2.6. Quantitative Assessment of RNA Extracts

RNA extracts underwent quantitative assessment via spectrophotometric measurement of RNA concentration (*Gene Quant II, Pharmacia*). The spectrophotometric assessment of extracts covered the absorbance measurement at the wave lengths of 230, 260, 280, and 320 mn, and then the A_260_/A_280_ ratio and protein content were determined for each sample. Absorbance value at the 260 nm wave length was used to calculate RNA concentration, based on the assumption that results of cuvette measurements using the 1 cm optical path equaling 1 OD_260_ correspond with the concentration of 40 mg RNA in 1 cm^3^ of extract.

### 2.7. Gene Expression Analysis with the Oligonucleotide Microarrays Technique

#### 2.7.1. General Method Assumptions

The material for the study consisted of total RNA extracted from the cell cultures with the TRIzol reagent (Invitrogen). The isolated RNA was purified with the RNeasy Total RNA Mini Kit and digested with DNase I (Qiagen) in order to eliminate any possible contamination with DNA. Approximately 8 *μ*g of total RNA was used for the double-stranded cDNA synthesis. Biotin-labeled cRNA was synthesized and biotinylated cRNA was then fragmented and hybridized with the Test3 microarray and HG U133A (Affymetrix) and labeled twice with the streptavidin-phycoerythrin conjugate and biotinylated streptavidin antibodies.

The fluorescence intensity was analyzed with the GeneArray Scanner G2500A. The quantity and quality of total RNA, cDNA, and cRNA were assessed spectrophotometrically and by means of the electrophoresis technique in 1% agarose gel.

#### 2.7.2. Purification of Total RNA Extract with RNasy Mini Kit

After a prior concentration measurement, 100 *μ*L of the RNA extract was obtained with RNase-free water. 350 *μ*L of RLT buffer with *β*-mercaptoethanol and 250 *μ*L of 96% ethanol were added, mixed by pipetting, and then applied to the column and centrifuged for 15 seconds at 12 000 r/min. The filtrate was discarded and the column was moved to a new tube and 350 *μ*L of RW1 was added and then centrifuged for 15 seconds at 10–12 000 r/min. For digestion, 70 *μ*L of RDD buffer was added to a tube with 10 *μ*L of DNase. This mixture was applied on the column membrane and left to digest for 15 min at room temperature. Then, 350 *μ*L of RW1 solution was added, the old flow-through was discarded, and the column was centrifuged for 15 sec. at 12 000 r/min. The flow-through was again discarded and 500 *μ*L of RPE solution was added to the column. The extracts were centrifuged for 1 min at 14 000 r/min; then the column was turned upside down and centrifuged again for the same time and rate. After centrifugation, the column was placed in a new tube and 30 *μ*L of H_2_O was added and was left for 10 min. Then the column was centrifuged and 20 *μ*L of H_2_O was added and left for a few minutes and then centrifuged again. After final cleanup, the RNA concentration in extracts was measured.

### 2.8. cDNA Synthesis

#### 2.8.1. First Strand cDNA Synthesis

In order to obtain the first strand of cDNA, 2 *μ*L 100 pM of T7 starter-oligo(dT)_24_(5′-GCCAGTGAATTGTAATACGACTCACTATAG GGAGGCGG-3′) was added to 11 *μ*L of RNA extract; the mixture was incubated at 70°C for 10 minutes and placed on ice. Then, 4 *μ*L of 5x First Strand Buffer, 2 *μ*L of 0.1 M DTT, and 1 *μ*L of 10 mM dNTPs (Invitrogen) were added. After 2 minutes of preincubation at 45°C, 1 *μ*L (200 U) of reverse transcriptase Supercscript II (Life Technologies) was added to the reaction mixture and incubation continued for another hour.

#### 2.8.2. Second Strand cDNA Synthesis

In order to obtain the second strand of cDNA, the following were added to the mixture obtained after the first strand synthesis: 30 *μ*L of 5x Second Strand Buffer, 91 *μ*L of RNase-free water, 3 *μ*L of 10 mM dNTPs, 4 *μ*L (40 U) of* E. coli* DNA Polymerase I, 1 *μ*L (10 U) of* E. coli* DNA Ligase, and 1 *μ*L (2 U) of Rnase H (Invitrogen). The mixture was incubated for 2 hours at 16°C.

Next, 2.5 *μ*L (10 U) of T4 DNA Polymerase I was added to the mixture and the mixture was incubated for another 5 minutes at 16°C. The composition of the reaction mixture used for the second strand cDNA synthesis is presented in Table 2. The reaction was stopped by adding 10 *μ*L of 0.5 M EDTA, and the double-stranded cDNA was extracted using the phenol/chloroform/isoamyl alcohol mixture. The aqueous phase was divided by means of Phase Lock Gel Light 1.5 mL tubes (Eppendorf). 0.5 volume of 7.5 M ammonium acetate (Sigma) and 2.5 volume of 96% ethyl alcohol were added to the aqueous phase, and it was left for 12 h at −20°C.

### 2.9. Purification of the Obtained cDNA

After freezing for the entire night at −20°C, 0.3 *μ*L of Palet Point (Merc) was added to the reaction mixture and it was centrifuged for 20 min at 12 000 r/min at room temperature; supernatant was discarded and the sediment was rinsed with 2x 70% ethanol (500 *μ*L) and centrifuged for 5 min at 12 000 r/min; the sediment was dried thoroughly and dissolved in 12 *μ*L of RNase-free water.

### 2.10. Biotinylated cRNA Synthesis

The template for the biotinylated cRNA synthesis consisted of 10 *μ*L ds cDNA, to which the following were added: 12 *μ*L of RNase-free water, 4 *μ*L of 2x hybridization buffer, 4 *μ*L of biotin-labeled ribonucleotides, 4 *μ*L of DTT, 4 *μ*L RNase inhibitor, and 2 *μ*L of T7 polymerase (*BioArray High Yield RNA Transcript Labeling Kit; Enzo*). The whole was left at 37°C for 5 h and mixed every 30 minutes.

### 2.11. Biotinylated cRNA Purification

After a 5-hour incubation, cRNA was cleaned up using 60 *μ*L of H_2_O, 350 *μ*L of RLT buffer, and 250 *μ*L of 96% ethanol added to the reaction mixture. The mixture was applied onto the RNAeasy column Total RNA Mini Kit (Qiagen) and was centrifuged twice for 15 seconds at 10 000 r/min. In the last stage 2x 20 *μ*L of H_2_O was added to the column membrane, it was centrifuged at 12 000 r/min, and then the cRNA concentration was measured.

### 2.12. Biotinylated cRNA Fragmentation

For fragmentation purposes, 16 *μ*g of cRNA and 8 *μ*L of fragmentation buffer (Affymetrix) were used, and the mixture was supplemented with up to 40 *μ*L of RNase-free water. After completion of this stage, the mixture was centrifuged and left for 35 min at 94°C and then placed on ice. The fragmentation result was checked on 1% agarose gel.

### 2.13. Assessment of cDNA, Biotinylated cRNA, and cRNA Synthesis after Fragmentation via Electrophoresis in Agarose Gel

Evidence of proper cDNA, biotinylated cRNA, and cRNA synthesis after fragmentation was assessed through electrophoresis in agarose gel with ethidium bromide. The resulting separation electrophoregram of the abovementioned products verified that samples were acceptable to use in the following analysis stage.

### 2.14. Hybridization of the Fragmented Biotinylated cRNA with Microarray

#### 2.14.1. Preparation of Hybridization Cocktail

In order to prepare the hybridization cocktail, 37.5 *μ*L of fragmented biotinylated cRNA was used and the following were added: 5 *μ*L of control oligonucleotide B2, 15 *μ*L of eukaryotic hybridization control (Affymetrix), 3 *μ*L of Herring Sperm (Invitrogen), 3 *μ*L of acetylated BSA (Invitrogen), 150 *μ*L of 2x hybridization buffer, and 86.5 *μ*L of RNase-free water. The cocktail was heated for 5 min at 99°C and for 5 min at 45°C and centrifuged for 5 min at 14 000 r/min.

#### 2.14.2. Application of Cocktail on Microarray

HG-U133A microarray (Affymetrix) was heated to room temperature and then 100 *μ*L of 1x hybridization buffer was applied. The chip was placed in hybridization oven for 10 min. Next, the buffer was removed from the chip and 200 *μ*L of hybridization cocktail was applied. The microarray was placed in the hybridization oven for 16 h at 45°C at 55 r/min.

#### 2.14.3. Washing and Scanning of Microarrays

After a 16-hour hybridization, the HG_U133A microarray was washed with buffer and stained with solutions (SAPE solution; streptavidin antibody solution) and then scanned with the GeneArray Scanner G2500A (Affymetrix).

### 2.15. Statistical Analysis

Data regarding expression levels obtained directly from the HGU133A array were reannotated with CDF control files developed by Dai et al. [16]. The next step consisted of microarray normalization with the RMA algorithm [17]. Three repetitions were replaced with an average value, with a prior detection of outliers by means of the Dean and Dixon method [18]. Determination of measurement noise threshold was conducted through empirical decomposition of probability density function of expression levels into normal components [19]. The component of the lowest mean value unchanged across all the microarrays was assumed to be the noise model. The threshold value of the filter was constructed at level 2.8 (in the field of logarithms to base 2). The mixture model of normal distributions constructed for the average SRL (*Signal Log Ratio*) of CD34^−^ cells from umbilical cord blood* versus* CD34^−^ of bone marrow was used to define the affinity of certain genes to subgroups of an increased, decreased, or unchanged value of expression level. Moreover, this mixture model was used to determine the FDR (*False Discovery Rate*) value for each of them.

In order to define qualitatively the processes in stem cells from umbilical cord blood in comparison with stem cells from bone marrow, an unsupervised gene clustering was conducted. Again, the Gaussian Mixture Model for empirical decomposition of expression levels constituted the algorithm enabling such a division. However, in this analysis additional limitations, based on the gene composition of each component that were the same across the experimental conditions, were added to the algorithm [20]. Clusters/classes obtained in this manner were compared to cellular processes in which they are involved. The categories of cellular processes were defined by gene ontology terms that are commonly applied for this purpose. A number of conditioning hypergeometric tests were conducted, verifying the one-sided hypothesis about overrepresentation. For each ontological term that was found to be significantly overrepresented statistically in a given gene cluster, the OR (*Odds Ratio*) value was determined.

Gene pathways whose components are genes belonging to specific clusters underwent an additional analysis, which was independent of a direct description of cellular processes. For this purpose we used data from the KEGG database. For each pathway the hypothesis of overrepresentation of a specified cluster of genes as compared with the whole scope of the analyzed genes was verified. Precise *p* values were determined.

Statistically significant values were those of FDR < 0.10 or *p* < 0.05.

## 3. Results

The first stage of the study consisted of data reannotation for expression levels through the CDF control files developed by Dai et al. [16]. For many years, the platform for gene expression profile was the Affymetrix GeneChip database. However, their probe section was created on the basis of prior gene and transcriptome annotations which significantly differ from the modern expertise. A comparative analysis between many popular Gene Chips and redefined probes based on updated gene data with consideration to genetic polymorphism showed a 30–50% discrepancy [16]; therefore it was decided to reannotate data as the first step in our study. The assessment of noise threshold was then completed, creating a mixture model of normal distributions for the mean SLR of CD34^−^ cells from umbilical cord blood versus CD34^−^ from bone marrow.

The next step comprised microarrays normalization through the RMA algorithm [17]. Three repetitions were replaced with a mean value, with a prior detection of outliers by means of the Dean and Dixon method [18]. Determination of the measurement noise threshold was conducted by empirical decomposition of probability density function of expression levels into normal components [19]. The noise model was assumed to be the component of the lowest mean value which was unchanged across all the microarrays. The threshold value of the filter was constructed at level 2.8 (in the field of logarithms to base 2).

**Figure 1:**
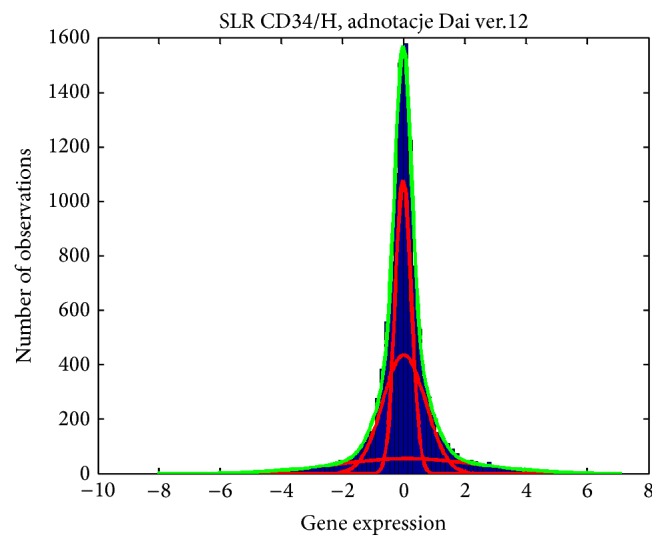
Mixture model of normal distributions (Gaussian Mixture Model) for a mean SLR (Signal Log Ratio) of CD34^−^ cells from umbilical cord blood* versus* CD34^−^ cells from bone marrow. Log (CD34-P)/( CD34-Sz).

As a result of the abovementioned procedures, a data set was obtained which described 11 979 genes which underwent further analysis. The first stage consisted of creating the mixture model of normal distributions constructed for a mean SLR (*Signal Log Ratio*) of CD34^−^ stem cells from umbilical cord blood* versus* CD34^−^ stem cells from bone marrow, which allowed defining the affinity criteria of specific genes to subgroups of an increased, decreased, and unchanged value of expression levels. This also enabled us to determine a FDR value (*False Discovery Rate*) for each of them (Figure 1).

This method is based on decomposition, that is, the histogram decomposition into (K)—Gauss's components as well as modeling the probability density function distributions (pdf) for given gene expression levels. The method was used for analysis of a histogram reflecting the frequency of genes of a specific expression level in the tested preparation. This initial analysis showed considerable similarity of gene expression of CD34^−^ cells from umbilical cord blood as compared with CD34^−^ cells from bone marrow.

### 3.1. Unsupervised Gene Clustering

During the next stage of data analysis a qualitative determination of the processes in CD34^−^ stem cells from umbilical cord blood in comparison with CD34^−^ stem cells from bone marrow was conducted using unsupervised gene clustering. Again, the Gaussian Mixture Model for empirical decomposition of expression levels constituted the algorithm enabling such a division. However, in this analysis additional limitations based on the gene composition of each component that were the same across the experimental conditions were added to the algorithm [20]. As a result, 15 gene clusters were obtained, two of which (numbers 9 and 13 in this study) contained discriminatory genes exhibiting a statistically significant increase (cluster number 9) and decrease of gene expression (cluster number 13) (Table 1).

The remaining clusters, differing in terms of the number of genes within the cluster, aggregated the genes of similar expression values. Differences between mean gene expression levels of CD34^−^ stem cells from bone marrow and CD34^−^ stem cells from umbilical cord blood in clusters numbered 1–8, 10–12, 14, and 15 are small or nonexistent. Thus, all these clusters were classified as the groups of genes exhibiting the same expression levels.

Cluster number 9 included 493 genes which show an increased activity in CD34^−^ stem cells from umbilical cord blood of the mean SRL (Signal Log Ratio) equaling 3.06. In contrast, cluster number 13 included a lower number of genes (*n* = 387) of decreased expression in CD34^−^ stem cells from umbilical cord blood for which SRL equals −3.43.

At the next stage, gene clusters/groups obtained in this manner were compared to biological processes in which they are involved, as defined by gene ontology (GO) terms that are commonly applied for this purpose. A number of conditioning hypergeometric tests were conducted, verifying the one-sided hypothesis about overrepresentation. For each ontological term that was found to be significantly overrepresented statistically in a given gene cluster, the OR (*Odds Ratio*) value was determined. All the clusters were classified through the GO classification system, that is, the clusters with genes discriminating both cell populations as well as the clusters grouping genes of a similar expression.

**Figure 2:**
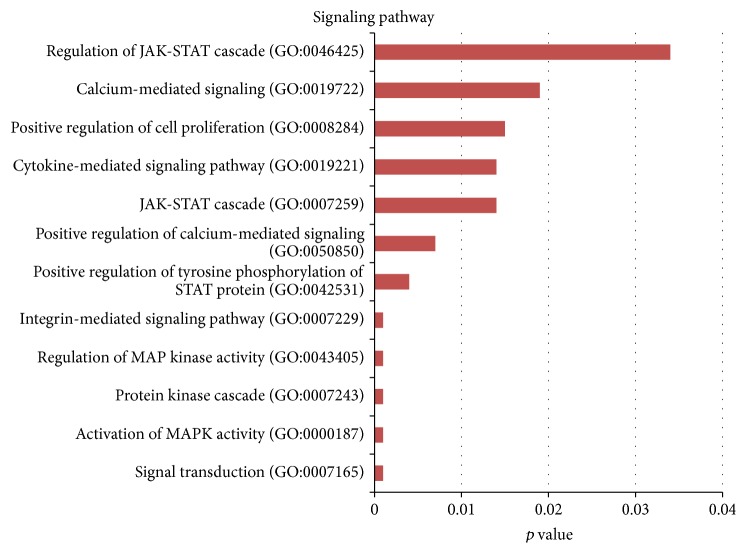
Functional annotations. 12 GO classification terms for biological processes in the group; signaling pathways in cluster 9 depending on the *p* value (*p* < 0.04).

Four hundred ninety-three genes of cluster 9, classified via the GO system, determined the terms of biological processes (*p* < 0.05) overrepresented in CD34^−^ stem cells from umbilical cord blood. The classification system determined 256 GO terms of biological processes, out of which GO terms of *p* value below 0.04 underwent further analysis. As a result of this analysis, the majority of GO terms represented only by one or two genes were rejected. The GO terms for biological processes in cluster 9 were assigned to one of six functional groups, including metabolism, biological processes regulation, cellular homeostasis, cellular defensive response and response to stimuli, cytokine production, and signaling pathways. The GO terms regarding signaling pathways are presented in Figure 2.

**Figure 3:**
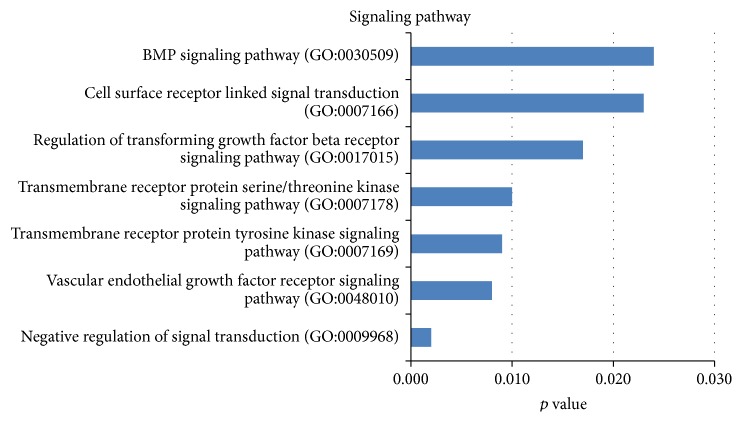
Functional annotations. 7 GO classification terms for biological processes in the group; signaling pathways in cluster 13 depending on the *p* value (*p* < 0.04).

The next stage consisted of a similar analysis of genes from cluster 13. GO system classification for 387 genes of cluster 13 determined 119 terms of biological processes (*p* < 0.05) of a decreased activity in CD34^−^ cells from umbilical cord blood as compared with CD34^−^ cells from bone marrow. The GO terms of *p* value below 0.04 underwent further analysis. As a result of this analysis, the majority of GO terms represented only by one or two genes were rejected. The GO terms for biological processes in cluster 13 were assigned to one of three functional groups, including metabolism, biological processes regulation, and signaling pathways. In CD34^−^ cells from umbilical cord blood as compared with CD34^−^ cells from bone marrow a decreased gene activity was observed of the BMP signaling pathway, TGF beta, transmembrane receptor of protein serine/threonine kinase, transmembrane receptor of tyrosine kinase, and VEGF (Figure 3).

Gene pathways whose components are genes belonging to specific clusters underwent an additional analysis, which was independent of a direct description of cellular processes. For this purpose data from the KEGG database (*Kyoto Encyclopedia of Genes and Genomes*) were used. For each pathway the hypothesis of overrepresentation of specific clusters of genes as compared with the whole scope of analyzed genes was verified, and precise *p* values were determined. For the purpose of this study, gene pathways for clusters 9 and 13 were analyzed because they contained genes discriminating both gene populations. On the basis of KEGG database, cytokine-cytokine receptor interaction pathway, toll-like receptor signaling pathway, epithelial cell signaling in* Helicobacter pylori* infection, and JAK-STAT signaling pathway are overrepresented in CD34^−^ cells from umbilical cord blood (Table 2).

In cluster 13, on the basis of KEGG classification, the CD34^−^ cells from umbilical cord blood show a decreased activity of focal adhesion signaling pathway, cell junctions, and TGF-beta signaling pathway (Table 3).

At the next stage, the genes of the remaining clusters numbered 1–8, 10–12, and 14 of a similar gene expression were analyzed. The analysis was based on the GO classification for each of the abovementioned clusters. The criteria used for choosing the clusters included the biological processes connected with cellular cycle as well as the processes connected with signaling pathways activity. A similar gene expression in CD34^−^ cells from umbilical cord blood and CD34^−^ cells from bone marrow was characteristic for such biological processes as cell division, mitosis, maintaining telomere endings, RNA and DNA processing, and a similar activity of Notch and Wnt signaling pathways genes.

An independent analysis was also carried out for genes of a similar activity in clusters 1–8, 10–12, and 14 on the basis of the KEGG database. CD34^−^ cells from umbilical cord blood and CD34^−^ cells from bone marrow showed similar activity of Wnt and Notch signaling pathways genes, p53 signaling pathway genes, cell cycle, PPAR, and mTOR (Table 4).

CD34^−^ cells from umbilical cord blood and CD34^−^ cells from bone marrow showed similar gene activity of Wnt and Notch signaling pathways both on the basis of the GO classification system and the KEGG database. Therefore, at the last stage of the study the activity of single genes of Wnt and Notch, as well as JAK-STAT signaling pathways chosen from 11976 sampled genes, was analyzed.

In accordance with the KEGG database, the JAK-STAT signaling pathway is defined by 155 genes, out of which there were 128 active genes in the studied material. Genes exhibiting increased activity in CD34^−^ cells from umbilical cord blood included SPRY2, CISH, CSF2, GRB2, IFNGR1, IL2RB, IL2RG, IL7R, IL10RA, IL15RA, PIM1, PTPN6, STAT1, STAT5A, and CCND3. Genes exhibiting decreased activity included PIAS3, IL11RA, and CBLB.

On the basis of the KEGG database, the Wnt signaling pathway is defined by 151 genes, out of which there were 132 active genes in the studied material. Genes exhibiting increased activity in CD34^−^ cells from umbilical cord blood include MMP7, PRKCB, PSEN1, RAC2, and CCND3. Genes exhibiting decreased activity include DKK1, WNT5A, WNT5B, FZD7, and TCF7L1.

On the basis of the KEGG database, the Notch signaling pathway is defined by 47 genes, out of which there were 38 active genes in the studied material. Genes exhibiting increased activity in CD34^−^ cells from umbilical cord blood included DTX4 and PSEN1.

## 4. Discussion

Nonhematopoietic stem cells were first identified by Friedenstein et al. who described the progenitor cells of rat bone marrow [21]. Currently, the nonhematopoietic stem cells, which are frequently referred to, although not quite correctly, as the mesenchymal stem cells, are isolated from various tissues, including the umbilical cord blood, placenta, and umbilical cord. A constantly increasing number of studies on MSCs have proved that in appropriate environmental conditions they are capable of differentiating into specialized mesodermal, endodermal, and even ectodermal cell lineages [2, 22–24]. In natural conditions they play an important role in cell regeneration processes and in repairing the tissues damaged by degenerating processes. Another intriguing characteristic of MSCs is their ability to avoid immunological identification and response; therefore they are referred to as the hypoimmunogenic cells. Despite great attention paid to mesenchymal stem cells, their biology is not yet fully understood. Introducing multipotential stem cells into clinical application requires a full understanding of the mechanisms which control key properties of the cell, such as cell self-replication versus differentiation and mobilization versus tissue homing. The understanding of these mechanisms must be based on strict definitions of human multipotential stem cells at the molecular level. In addition, understanding of molecular mechanisms of self-replicating, pluripotentiality, plasticity, and differentiation of stem cells not only is a necessary condition to overcome limitations of stem cells application as a therapeutic factor but also is key to understand a fundamental mystery of the development of human organism.

This paper presents a comparison of gene activity of the nonhematopoietic cells of umbilical cord blood and bone marrow using oligonucleotide microarrays. Gene activity was tested in stem cells CD34 and included 11 979 genes obtained after completing procedures to increase the purity of cell samples. Cell populations included in the study were also limited based on the noise threshold revealed in the final comparative analysis. The gene expression profiles of the compared populations of CD34^−^ stem cells from umbilical cord blood and CD34^−^ stem cells from bone marrow were very similar (Figure 1). For a better illustration of the biological molecular activity and the activity of signaling pathways in the studied cell populations, a transcription profile analysis with the GO and KEGG databases was completed. In terms of fundamental signaling pathways for biological activity of stem cells like JAK-STAT, Wnt, and Notch we recorded discrete differences among expressed genes from umbilical cord blood cells as compared to bone marrow cells.

The analysis regarding signaling pathways showed increased activity of the JAK-STAT pathway in CD34^−^ stem cells from umbilical cord blood both on the basis of the gene ontology classification and the KEGG database (Figure 2 and Table 2). The increased activity regarded 15 genes which take part in the JAK-STAT cascade, that is, SPRY2, CISH, CSF2, GRB2, IFNGR1, IL2RB, IL2RG, IL7R, IL10RA, IL15RA, PIM1, PTPN6, and CCND3, including the genes which code transcription factors STAT1 and STAT5A. In previous publications the significance of the JAK-STAT signaling pathway is best described in the embryonic and hematopoietic stem cells. Ramalho-Santos et al. searched for a transcription profile describing both the embryonic stem cells and the stem cells of an adult organism on the basis of the study of embryonic cells, neural stem cells, and hematopoietic stem cells activity [25]. These studies attempted to create a molecular signature of the stem cells in relation to the self-regeneration and differentiation abilities of the cells. The active JAK-STAT pathway belongs to the basic attributes defining the so-called “stemness” phenomenon. In the embryonic stem cells the JAK-STAT signaling pathway promotes proliferation in the form of self-replication. The activating factor LIF (*Leukemia Inhibitory Factor*) provokes phosphorylation and dimerisation of STAT3, which after translocation to the cell nucleus stimulates the expression of gene group responsible for self-replication of the embryonic stem cell. However, the JAK-STAT signaling pathway is not the only factor responsible for proliferation regulation of the embryonic stem cells. Internal processes regulating the multiplication processes independent of the STAT3 activity, such as the Nanog and Oct-4 activity, are also important [15, 17, 26, 27]. In our study material, the Nanog and Oct-4 genes were not active in both types of nonhematopoietic stem cells, which is in agreement with other publications devoted to this subject [28, 29]. The STAT1 transcription factor, overactive in our study in CD34^−^ cells of umbilical cord blood, is responsible, among others, for the expression of interferon-activated genes involved in cell defense against pathogenic factors [30]. Moreover, it is believed that STAT1 is a medium in expression of gene group which is important for cell viability in response to stimulating factors. In 2006 Kim's team described STAT1 as a highly overactive gene in human mesenchymal stem cells [31]. In comparison, the transcription factor STAT5, which functions in the form of two isoforms STAT5a and STAT5b, is involved in the cellular proproliferation responses and antiapoptosis processes [32, 33]. STAT5 is activated by various cytokine receptors. In our study, the isoform STAT5A showed increased activity. It is believed that the STAT5 factor activates specifically the cellular cycle genes, that is, cyclins [34, 35]. In human stem cells the STAT factors activate the expression of cyclin D which codes the regulating subunit of the CYCD/CDK4 complex driving the G1/S phase transition, which in turn activates the cell cycle progression [32, 36]. STAT5a and STAT5b also play an important role in expression of genes promoting hematopoietic stem cells survival [37]. Gene expression profiling over a range of STAT5 activities in human CB cells revealed subsets of genes that are associated with the self-renewal and long-term expansion phenotype. Taking into account the fact that in STAT5^−/−^ mice myelopoiesis appears to be relatively unaffected it can be assumed that STAT5 can play a key role in assessing the quality of cells [38]. Additionally, not only is the JAK-STAT signaling pathway a significant signal transduction component which controls cell proliferation and purpose, but it also regulates cell migration, which is important both in the primary processes of gastrulation and in the tissue regeneration. For instance, the JAK-STAT signaling pathway is connected with cardioprotection [39] as well as with processes of regeneration and remodeling of skeleton [40, 41]. The active JAK-STAT signaling pathway helps to regulate cell organization within cell grouping [42]. The above results may suggest a greater self-renewal potential of CD34^−^ stem cells of umbilical cord blood, long-term expansion phenotype, and their higher ability in terms of tissue regeneration.

The Wnt signaling pathway is perceived as a signaling cascade involved in the control of fundamental processes of nondifferentiated mesenchymal stem cells, such as cell reproduction and directed differentiation. Both methods of gene activity classification, that is, GO and KEGG, showed a similar activity of the majority of Wnt signaling pathway genes (Table 4). In addition, the analysis of separate genes taking part in the Wnt signaling pathway and coding 19 glycosylated molecules of extracellular signaling of the Wnt family, cellular surface receptors including Frizzled receptors, intracellular signaling molecules and genes involved in cell cycle and taking part in regulation of proliferation, growth and modification of proteins, showed that the expression profile was very similar, except for single genes of an increased and decreased expression in CD34^−^ stem cells of umbilical cord blood in our study, that is, MMP7, PRKCB, PSEN1, RAC2, and CCND3, respectively, as well as DKK1, WNT5A, WNT5B, FZD7, and TCF7L1. The Wnt5a ligand is involved in the noncanonical Wnt signaling pathway transmission and it is assumed that it may inhibit the canonical Wnt pathway in mouse embryonic cells similarly to Dkk1. The role of the noncanonical Wnt signaling pathway, which in our results seemed to prevail in CD34^−^ stem cells of bone marrow, is not clear. It is believed that the noncanonical Wnt signaling pathway controls the reorganization of cytoskeleton muscle proteins, tissue polarity, and cell movement [43]. In contrast, the canonical signaling pathway, which activates target gene transcription through the increase of beta-catenin concentration, seems to be more active in CD34^−^ stem cells from umbilical cord cells. Its role is to control the tissue-specific cell purpose during embryogenesis as well as regulation of proliferation in adult tissues [44]. Additionally, high activity of WNT5A gene is noticeable in the human MSCs differentiation into osteoblasts, as well as in early stages of adipogenesis [45, 46]. According to the publication of Yang et al., the Wnt5a and Wnt5b glycoproteins are also responsible for coordination of chondrocyte proliferation and differentiation [47]. Wnt5a is believed to be an important factor taking part in the positive autocrinic regulation of HSC repopulation ability [48]. In their publication, Prockop et al. suggest that a high WNT5A expression in MSCs is characteristic for the end of the exponential and stationary growth phases in cell cultures in* in vitro* conditions [49]. Slight changes in the Wnt gene activity, especially in WNT5A activity, may result from the role played by MSCs in their environmental niche. Bone marrow MSCs seem to be more involved in the renewal processes of the progenitor cells pool which reside in bone marrow. The researches show that the expression of Wnt ligands and Fz receptors in MSCs shows some correlation of Wnt/Fz pairs, such as Wnt5a/Fz5 [50]. The above discussed study results showed that there was a coexpression of WNT5A/FZD7. Their higher expression was characteristic for the bone marrow cells, while the previously published data suggested differences in Wnt/Fz activity depending on the origin-mouse versus human MSCs [51]. Currently available data suggest differences which may be characteristic not only for proliferation and differentiation programs but also for the phenotype of MSCs from umbilical cord blood and bone marrow, which is in agreement with the results published by Baksh et al. [7]. The remaining genes take part in the Wnt signaling cascade of increased activity in CD34^−^ from umbilical cord blood, such as MMP7 belonging to metalloproteinases, which play a role in the extracellular array remodeling and wound healing [5].

The Notch signaling pathway belongs to the processes of intercellular communication which regulate gene expression patterns defining a dualistic character of stem cells reflected by the asymmetric cell division [52]. Additionally, the Notch signaling cascade controls various cells biological processes, not only proliferation and differentiation but also apoptosis and regeneration. The signaling activity of Notch pathway genes determined in the study of 38 genes showed their similar expression as defined by the KEGG classification (Table 4). A similar activity level was noted for all four Notch 1–4 receptors, as well as for JAG1 and JAG2 ligands. The differences were related to two genes DTX4 and PSEN1, which were overactive in CD34^−^ cells from umbilical cord blood. The function of the PSEN1 gene is mainly developmental processes regarding cell differentiation during neurogenesis. And the lack of this gene leads to a premature directed differentiation of neural progenitor cells [53]. The activity of the Notch gene cascade plays an important role at all levels of stem cell development, starting from the embryonic stem cell through fetal cells to adult organism stem cells. Notch is significantly involved in the asymmetric segregation of factors determining cell purpose during replication divisions of the stem cell pool. The significance of receptors and ligands is best described in the hematopoietic stem cells. Expression of the constitutively active Notch1 gene in hematopoietic stem cells induces the immortalized cell lineages which can reproduce the myeloid and lymphoid cell lineage in a long-term repopulation in animal studies [54, 55]. Apart from that, it delays differentiation of human hematopoietic progenitor stem cells* in vitro* [56]. The studies suggest that Notch1 is an important factor in HSC self-renewal and can also participate in the inhibition of stem cell differentiation. The scientific findings reveal that a suppression of the Notch signaling pathway via deletion of key genes in HSC did not cause cells to lose their ability of long-term hematopoiesis renewal in animal models [57]. Therefore, the role of ligands and Notch receptors in HSC has not been precisely defined yet, and current publications do not provide precise data regarding the role played by particular Notch signaling pathway genes in mesenchymal stem cells. The only phenomenon which appears to be superior and characteristic for all stem cells, including MSCs, is the significance of the Notch cascade in asymmetrical cell divisions. This is a significant component of stem cells population self-renewal, as well as the functionality of the cascade in the stem niche. This phenomenon constitutes an integral part of the hypothesis about stem cell population aging. However, further investigation will be required to determine if the differentially expressed genes described in the studies above could serve as stemness genes.

Remaining signaling pathways which revealed increased activity in the studied CD34^−^ cells of umbilical cord blood included MAPK (*Mitogen-Activated Protein Kinase*, Figure 2) which regulates a wide range of cell biological activities, such as gene expression, mitosis, proliferation, differentiation, migration and apoptosis processes [58]. The MAPK pathways take part in regulation of early embryonic development and in defining the role of embryonic stem cells from the early developmental phases until the moment of formation of differentiated adult cells. However, this precise role played by the MAPK pathways in various stem cell types has not yet been defined.

Transmission via calcium ions was also singled out in the group of the overactive signaling pathways of the studied CD34^−^ stem cells of umbilical cord blood versus CD34^−^ stem cells of bone marrow (Figure 2). The ions are a vital and commonly present mediator in the process of extracellular signals transmission and conversion into a cellular response. Variations of calcium ions concentrations regulate gene expression through an impact on the activity of numerous signaling pathways. Intracellular increase of calcium ions concentration also regulates cell activities, including adhesion, motility, gene expression, and proliferation [59]. The significance of transmission via calcium ions and the ways of their concentration changes in mesenchymal stem cells still have not been studied. Kawano et al. were the first to report that in the nondifferentiated MSCs there are spontaneous changes of calcium ions concentrations despite the lack of any stimulating factors [60]. As reported, the calcium ion transmission through the activation of different gene groups may direct the cells onto specific developmental pathways [61].

The integrin-mediated signaling pathway takes part in extracellular array remodeling, which means that it controls cell behavior and tissue organization. Its increased activity was observed in our study in CD34^−^ stem cells of umbilical cord blood (Figure 2). In recent years the role of integrins and selectins has been stressed in the adhesion and transendothelial migration, which has a significant meaning in the processes of repopulation of tissues damaged by diseases and their regeneration via the circulating MSCs. For instance, Ip et al. showed that blocking of beta 1-integrin, which constitutes a component of the adhesive molecule VLA-4, in MSC weakened the ability of mesenchymal stem cells to engraft into the infarction-changed myocardium [62]. The above results may suggest a better migration ability of CD34^−^ stem cells of umbilical cord blood into the area of pathologic tissue. MSCs own cytokinetic activity that has similar significance in terms of tissue regeneration. The study of cytokinetic activity of CD34^−^ stem cells of umbilical cord blood showed increased activity of signaling pathways activated via cytokines (Figure 2; Table 2). On the other hand, the TLR signaling pathway (*toll-like receptor signaling*), which in our own results revealed a significant increased activity in CD34^−^ stem cells of umbilical cord blood (Figure 2), increases the immunosuppressive characteristics of MSCs. Weakening of the immunological response generated by the TLR pathway depends on the production of immunosuppressive kynurenines by the indoleamine 2,3-dioxygenase enzyme which degrades tryptophan. Induction of indoleamine 2,3-dioxygenase by TLR entails autocrine signaling loop of interferon-beta, which plays a role in MSCs immunomodulative effects [63].

According to the GO and KEGG classifications carried out for cluster 13 with the gene pool of a decreased expression, a statistically significant decrease of gene activity was shown for the TGF-beta and BMP pathways (Figure 3; Table 3). It is believed that the family of TGF-beta members, which also includes BMP, plays an important role in initiating the process of a directed MSCs differentiation. TGF-beta signaling transmission runs through specific serine-threonine kinase receptors and their nuclear receptors called the Smad proteins. TGF-beta signaling transmission promotes the process of early differentiation stages into the chondrogenic and osteogenic lineages [64, 65]. However, cell differentiating into the adipogenic lineage results in inhibition of the TGF-beta signaling pathway. This was confirmed in experimental studies developed by Ng et al. where the inhibition of TGF-beta transmission resulted in an increase of adipogenesis and a decrease of differentiating ability towards chondrocytes [66]. The BMP signaling pathway strongly promotes differentiation towards osteoblasts, but it also leads to inhibition of myogenesis and adipogenesis. Such a gene expression profile may suggest that the MSCs biological characteristics are strongly influenced by the significance of stem cell niche. Therefore the mesenchymal stem cells from bone marrow may be more involved in the promotion of the phenomena which maintain the stability of bone marrow environment.

## 5. Conclusions 

Multipotentiality of the mesenchymal stem cells, their high proliferation ability, and not too-demanding culturing conditions reveal their potential for wide application in various medical areas. These are the reasons for the common pursuit to understand their biology based on more and more sublime biomolecular procedures. Scientists have been searching for an unequivocal marker which would characterize the mesenchymal stem cells and simultaneously would enable a separation of a homogenous MSC population. Understanding of molecular mechanisms controlling self-replication and differentiation is becoming a fundamental issue for clinical application of MSC. In this respect, the activity of signaling pathways is important, yet the interpretation of the pathways, especially their interdependence, constitutes a great challenge for the scientific world. Moreover, the answer to the question regarding MSC innidation and its survival ability in host tissues after transplantation is yet to be found. Explanation of the abovementioned issues will require a more global understanding of biological activity of these cells, especially on the basis of the significance of the mesenchymal niche environment of stem cells and its impact on the gene and epigenetic activity. It should be assumed that continued progress in the understanding of MSCs will finally lead to their common therapeutic and preventive application and consequently to the improvement of the quality and length of human life.

## Figures and Tables

**Table 1:** 15 clusters obtained as a result of the unsupervised gene clustering. Mean CD34^−^ BM (mean of CD34^−^ stem cells from bone marrow). Mean CD34^−^ UCB (mean of CD34^−^ stem cells from umbilical cord blood). *N* (a number of genes in a cluster). SRL (Signal Log Ratio).

Cluster	Mean CD34^−^ BM	Mean CD34^−^ UCB	SD CD34^−^ BM	SD CD34^−^ UCB	*N*	*p* value	Mean SLR	−95% CI SLR	+95% CI SLR	Conclusion
1	4.97	4.91	0.08	0.09	1102	0.00000023	−0.06	−0.08	−0.04	No change
2	11.57	11.49	0.35	0.47	54	0.26488907	−0.08	−0.22	0.06	No change
3	8.02	8.05	0.30	0.32	592	0.63190010	0.01	−0.05	0.07	No change
4	4.45	4.40	0.06	0.05	999	0.00000001	−0.06	−0.08	−0.04	No change
5	7.02	7.14	0.24	0.21	898	0.00000009	0.11	0.07	0.16	No change
6	5.54	5.50	0.12	0.13	1133	0.00000889	−0.06	−0.09	−0.03	No change
7	3.53	3.46	0.04	0.05	960	4.51861*E* − 13	−0.07	−0.09	−0.05	No change
8	4.76	4.83	0.71	0.88	961	0.03102164	0.11	0.01	0.20	No change
**9**	5.64	7.15	1.58	2.70	493	**p** < 0.0000001	3.06	2.95	3.16	**Up**
10	3.11	3.09	0.04	0.04	872	0.00325256	−0.03	−0.05	−0.01	No change
11	2.76	2.78	0.02	0.02	647	0.00002947	0.03	0.01	0.04	No change
12	3.98	3.90	0.06	0.07	1133	5.44009*E* − 15	−0.08	−0.10	−0.06	No change
**13**	7.39	4.86	2.85	1.01	387	**p** < 0.0000001	−3.43	−3.57	−3.29	**Down**
14	6.21	6.21	0.26	0.21	1353	0.44902052	−0.01	−0.05	0.02	No change
15	9.30	9.36	0.64	0.84	395	0.15369595	0.08	−0.03	0.20	No change

**Table 2:** Gene pathways based on the KEGG classification of an increased expression in CD34^−^ stem cells from umbilical cord blood.

KEGGID	*p* value	Odds ratio	Exp. count	Count	Size	Term
4060	0.001	2.947	14	33	223	Cytokine-cytokine receptor interaction
4620	0.001	3.672	6	17	92	Toll-like receptor signaling pathway
5120	0.001	3.748	4	12	63	Epithelial cell signaling in *Helicobacter pylori* infection
4630	0.012	2.067	8	15	130	JAK-STAT signaling pathway

**Table 3:** Gene pathways based on the KEGG classification of a decreased expression in CD34^−^ stem cells from umbilical cord blood.

KEGGID	*p* value	Odds ratio	Exp. count	Count	Size	Term
4510	0.001	7.517	7	33	188	Focal adhesion
1430	0.001	8.157	4	22	109	Cell junctions
4350	0.001	4.038	3	10	82	TGF-beta signaling pathway

**Table 4:** Gene pathways based on the KEGG classification of a similar expression in CD34^−^ stem cells from umbilical cord blood and CD34^−^ cells from bone marrow for clusters 1–8, 10–12, and 14-15.

KEGGID	*p* value	Odds ratio	Exp. count	Count	Size	Term
04310	0.018	1.880	10	17	131	Wnt signaling pathway
04330	0.025	2.722	3	7	39	Notch signaling pathway
04115	0.038	2.157	5	9	61	p53 signaling pathway
04110	0.009	2.028	10	18	100	Cell cycle
03320	0.020	2.791	3	7	60	PPAR signaling pathway
04150	0.035	2.220	5	9	46	mTOR signaling pathway
